# Exosomes in gastric cancer: roles, mechanisms, and applications

**DOI:** 10.1186/s12943-019-1001-7

**Published:** 2019-03-15

**Authors:** Min Fu, Jianmei Gu, Pengcheng Jiang, Hui Qian, Wenrong Xu, Xu Zhang

**Affiliations:** 1 Institute of Digestive Diseases, The Affiliated People’s Hospital of Jiangsu University, Zhenjiang, 212002 Jiangsu China; 2 Jiangsu Key Laboratory of Medical Science and Laboratory Medicine, School of Medicine, Jiangsu University, 301 Xuefu Road, Zhenjiang, 212013 Jiangsu China; 3 Departmemt of Clinical Laboratory Medicine, Nantong Tumor Hospital, 30 Tongyang North Road, Nantong, 226361 Jiangsu China

**Keywords:** Exosomes, Gastric cancer, Progression, Biomarker, Target

## Abstract

Exosomes are nanosized extracellular vesicles that can be released by almost all types of cells. Initially considered as the garbage bins acting to discard unwanted products of cells, exosomes are now recognized as an important way for cellular communication by transmitting bioactive molecules including proteins, DNA, mRNAs, and non-coding RNAs. The recent studies have shown that exosomes are critically involved in human health and diseases including cancer. Exosomes have been suggested to participate in the promotion of tumorigenesis, tumor growth and metastasis, tumor angiogenesis, tumor immune escape, and tumor therapy resistance. Increasing evidence indicate that exosomes play important roles in gastric cancer development and progression. In this review, we summarized the current understanding of exosomes in gastric cancer with an emphasis on the biological roles of exosomes in gastric cancer and their potential as biomarkers for gastric cancer diagnosis as well as potential targets for gastric cancer therapy.

## Background

Gastric cancer (GC) ranks as the fifth most frequently diagnosed cancer and the third leading cause of cancer death worldwide [1]. Despite recent advances in therapeutic methods including surgery combined with chemotherapy and radiotherapy, the prognosis for advanced GC patients remains very poor. The early stage of GC often remains asymptomatic, leading to delayed diagnosis and missed opportunity of radical operation. Therefore, early diagnosis for resectable GC is essential for increasing the long-term survival of patients, which highlights the requirements for discovering novel noninvasive biomarkers with high sensitivity and specificity for the screening of early GC.

The past decade has witnessed a renewed interest of exosomes. These nanoscale vesicles of endocytic origin are secreted by almost all types of cells. Exosomes range 30–150 nm in diameter and have a buoyant density of 1.10–1.14 g/mL [2, 3]. Exosomes are composed of a lipid bilayer containing transmembrane proteins and enclosing cytosolic proteins, lipids, and nucleic acids. Stuffed with various biomolecules, exosomes are able to mediate local and distant cell communication through transferring specific cargos, during both physiological and pathological conditions [4, 5]. The exosomal cargos could reflect the identity of the originated cells. Moreover, with the lipid bilayer structure properly protecting the contents from degradation, exosomes exist stably in various biological fluids (e.g. blood, urine, saliva, and cell culture media) [6–9]. These unique properties make exosomes a potential candidate as reliable biomarkers. As increasingly exemplified in the literature, exosomes have become an important mediator of tumorigenesis, tumor growth, angiogenesis and metastasis [10]. Exosomes and their derived cargos have been applied as novel biomarkers for cancer diagnosis and prognosis. In this review, we highlight and discuss about the relationship between exosomes and GC, with a special focus on their roles, mechanisms of actions, and potential clinical application values as biomarkers and therapeutic targets in GC.

## The biological properties and isolation of exosomes

**Fig. 1:**
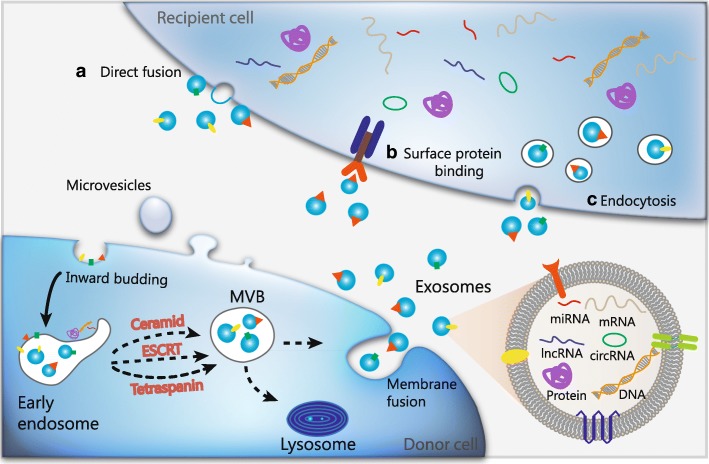
The biogenesis and contents of exosomes. The inward budding of the plasma membrane leads to the formation of early endosomes with membrane proteins incorporated. Then the invagination of endosomes and the enclosing of selected cargos including nucleic acids and proteins results in the generation of multivesicular bodies (MVBs), through either ESCRT-dependent or ESCRT-independent mechanisms. Subsequently, these MVBs fuse with plasma membrane and release exosomes into extracellular place. Exosomes release these cargos (proteins, mRNAs, miRNAs, lncRNAs, circRNAs and DNAs) to the recipient cells via mechanisms including a) direct fusion, b) binding with surface proteins, and c) endocytosis

Exosomes had been initially dismissed as cellular “debris” since first described as vesicles of endosomal origin secreted from reticulocytes in the 1980s [11]. More than a decade later, Raposo and coworkers reported that exosomes isolated from Epstein-Barr virus-transformed B lymphocytes were antigen-presenting and could induce T cell responses [12]. Since then, the research interest around exosomes increased substantially as they appeared to participate in cellular processes. The biogenesis of exosomes starts with an inward budding of the plasma membrane, resulting in the formation of early endosomes with membrane proteins incorporated (Fig. 1). With the invagination of endosomes and the enclosing of selected proteins and RNAs, multivesicular bodies (MVBs) are further generated. Subsequently, these MVBs fuse with plasma membrane and release exosomes into extracellular place [13]. Different mechanisms have been proposed to regulate the formation of MVBs. Among them, the hitherto best-described mechanism is driven by the endosomal sorting complex required for transport (ESCRT), which is composed of approximately thirty proteins that assemble into four complexes (ESCRT-0, −I, −II and -III) with associated proteins conserved from yeast to mammals [14]. Besides, ESCRT-independent mechanisms for exosomal formation have also been proposed, with pathways depending on ceramide [15] and tetraspanins [16]. However, numerous unanswered questions still remain in this field which need further investigation.

Different methods have been developed to isolate exosomes based on their biophysical and biochemical properties, including ultracentrifugation-based techniques (differential ultracentrifugation and density gradient ultracentrifugation), size-based techniques (ultrafiltration and size exclusion chromatography), immunoaffinity capture-based techniques (microplate-based magneto-immunocapture), exosome precipitation and mircrofluidics-based techniques (acoustic nanofilter and porous silicon nanowire-on-micropillar structure) [17, 18]. Differential ultracentrifugation is currently considered as the gold standard of exosome isolation, easy to use with large sample capacity and high exosome yields. Although widely used, it is a cumbersome method, laborious and time-consuming, with requisite expensive equipment. Moreover, exosomes isolated by using differential ultracentrifugation often contain protein aggregates and the final ultracentrifugation step may damage exosomes, thus impeding downstream analysis [19]. Likewise, none of the existing methods leads to a perfect isolation of pure exosomes. Size exclusion chromatography has a relatively high yield but is hard to scale up. Immunocapture is suitable for the isolation and subtyping of specific exosomes, yet costly with low capacity and low yields, and only works with cell-free samples. Exosome precipitation assays are fast with high recovery but co-precipitate contaminants like proteins. Combination of different isolation methods like ultracentrifugation and immunoaffinity capture may achieve some improvements but the additional work flows and higher costs need to be taken into consideration [18].

Certain proteins enriched in exosomes are often used as markers for exosomal identification, such as tetraspanins (CD63 and CD81), heat shock protein 70 (HSP70), the ESCRT-III binding protein ALG2-interacting protein X (ALIX), tumor susceptibility gene 101 protein (TSG101) and major histocompatibility complex (MHC) molecules [20, 21]. However, the indeed specificities of these protein markers for certain exosomal subgroups still need to be verified by further proteomic studies. Importantly, isolation methods should be well controlled in this process since they may yield exosomal subgroups of variable heterogeneity [16]. Recently, unbiased deep sequencing has revealed that exosomes contain RNAs including mRNA, miRNA and an array of other non-coding RNAs, many of which are closely associated with the originated cells by selective incorporations into exosomes. On the other side, less attention has been paid on the RNAs which are ubiquitous among all exosomes due to their specific targeting into MVBs during biogenesis [22]. A series of RNAs such as U6 snRNA, miR-191-5p and miR-16 have been widely utilized as internal controls during the analysis of exosomal non-coding RNAs [23–25]. Notably, the variation in exosomal RNA yield and patterns brought by different RNA isolation methods should be strictly controlled in the application of these exosomal RNAs as internal controls [26].

## The roles of exosomes in GC

**Fig. 2:**
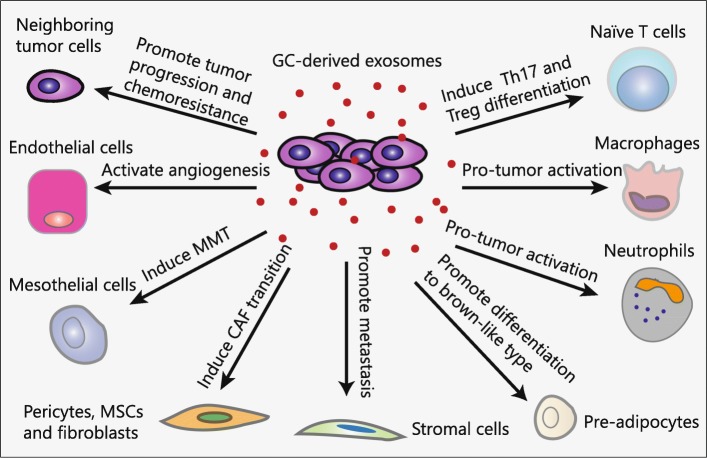
Roles of tumor cells derived exosomes in GC. Exosomes are critically involved in GC progression including tumorigenesis, metastasis, angiogenesis, immune evasion and drug resistance by transferring functional biomolecules. GC cells derived exosomes can modulate immunity by activating pro-tumor phenotypes of neutrophils and macrophages and inducing the differentiation of T cells to Th17 and Treg cells. GC cells derived exosomes can convert pericytes, fibroblasts and MSCs into myofibroblasts to facilitate tumor angiogenesis and metastasis. Moreover, GC cells derived exosomes can activate endothelial cells to support tumor angiogenesis and promote significant adhesion between mesothelial and GC cells. GC cells derived exosomes can help to create a favorable microenvironment for liver metastasis by acting on liver stromal cells. In addition, pre-adipocytes prefer to differentiate into brown-like type by GC cells derived exosomes

Emerging evidence indicate that exosomes are critically involved in GC progression including tumorigenesis, metastasis, angiogenesis, immune evasion and drug resistance (Fig. 2). Qu et al. first described the role of exosomes in GC in 2009. They reported that GC cell-derived exosomes promoted GC cell proliferation by activation of PI3K/Akt and MAPK/ERK pathways [27]. The following studies also supported the hypothesis that exosomes promote GC cell growth in an autocrine manner [28]. The pre-exposure of GC cells to their derived exosomes resulted in enhanced tumor growth and angiogenesis in the NOD/SCID mouse model, suggesting a pro-tumor role of exosomes as macro-messenger by delivering signals and molecular cargos [29]. Additionally, Li and colleagues found that exosomes from gastric cancer cells significantly increased gastric cancer cell proliferation and invasion. Further investigation unveiled that CD97 was involved in exosomes-mediated promotion of GC cell proliferation and invasion through activation of MAPK signaling pathway [30]. Besides, CD97 was proved to be exosome-dependent and played a pivotal role in this process [31].

Exosomes can mediate GC metastasis to local or distant tissues and organs. A pioneering study by Arita and collaborators suggested that the internalization of GC cells derived exosomes into mesothelial cells promoted significant adhesion between mesothelial and gastric cancer cells by enhancing the expression of fibronectin 1 and laminin gamma 1 [32]. Subsequently, Tanaka and colleagues demonstrated that the incorporation of GC derived exosomes induced peritoneal mesothelial cell (PMC) infiltration, which in turn accelerated tumor invasion in the gastric wall, and PMC-led cancer cell invasion in disseminated tumors within the abdominal wall and diaphragm [33]. This hypothesis was subsequently strengthened by Deng and coworkers [34], with the description that GC cells derived exosomes induced injury of PMCs through apoptosis and mesothelial-to-mesenchymal transition (MMT), resulting in mesothelial barrier destruction and peritoneal fibrosis. These findings support that exosomes play a crucial role in remodeling the premetastatic microenvironment and mediating peritoneal metastasis. Moreover, Miki et al. demonstrated that CD9-positive exosomes derived from cancer-associated fibroblasts (CAFs) stimulated the migration and invasion ability of scirrhous-type gastric cancer cells [35], indicating that exosomes from the stromal cells in the tumor microenvironment could also support GC metastasis.

Another important feature of GC-derived exosomes is their ability to modulate tumor immunity. A previous study by Qu et al. in 2009 showed that gastric cancer exosomes induced Jurkat T cell apoptosis in a time- and dose-dependent manner, which was regulated by Cbl family of ubiquitin ligases through inactivating PI3K/Akt signaling and mediating caspase activation [36]. In a latter study, Wu and colleagues suggested that exosomes derived from gastric cancer cells stimulated the activation of NF-κB pathway in macrophages to acquire a proinflammatory phenotype, resulting in the enhancement of tumor cell growth, migration, and invasion [37]. More recently, Wang et al. reported that exosomes derived from gastric cancer cells can effectively induce the production of PD-1^+^ tumor-associated macrophages (TAMs), which interacts directly with PD-L1^+^ cells to produce IL-10, resulting in dysfunction of CD8^+^ T cell and favorable conditions for GC progression [38]. More recently, Zhang et al. demonstrated that GC cells derived exosomes could induce neutrophils to polarize to N2 tumor-associated neutrophils (TAN), thus promoting gastric cancer cell migration [5].

Exosomes can also mediate drug resistance in GC. Ji et al. reported that mesenchymal stem cells (MSCs) derived exosomes could induce the resistance of GC cells to 5-fluorouracil (5-FU), which may be associated with its role in activating Ca^2+^/Raf/MEK/ERK signaling pathway and upregulating the expression of multidrug resistant proteins in GC cells [39].

## The functions of exosomes-derived cargos in GC

The bioactive molecules shuttled by exosomes lead to the exchange of genetic information between cells and the reprogramming of recipient cells. The contents of exosomes have been identified in multiple organisms and are shared in free databases such as ExoCarta (www.exocarta.org), Vesiclepedia (http://www.microvesicles.org) [40], and Evpedia (http://evpedia.info) [41]. The latest version of ExoCarta shows that there are 41,860 protein entries, 4946 mRNA entries, and 1116 lipid entries in exosomes from 286 independent studies. These exosomal cargos can help to elucidate their biogenesis process, identify their originated cells and mediate their biological functions [42]. The knowledge of exosomal contents in gastric cancer is still at its early stage. In the following section we will discuss about the various contents in exosomes from gastric cancer cells and other cells in tumor microenvironment, including exosomal proteins, miRNAs, lncRNAs and circRNAs (Table 1).

**Table 1:** Overview of exosomal cargos and functions in GC

Cargo type	Exosomal cargo	Originated cells	Recipient cells	Function	Reference
Protein	BMPs	SGC-7901	Pericytes	Induce transition into cancer-associated fibroblasts	[45]
	EGFR	SGC-7901	Primary mouse liver cells	Promote gastric cancer liver metastasis	[46]
	TGF-β1	Plasma from GC patients	CD4^+^CD45RA^+^ naïve T cells	Induce Treg cell differentiation	[43]
	TGF-β	SGC-7901 and HGC-27	HucMSCs	Trigger differentiation to carcinoma-associated fibroblasts	[44]
	HMGB1	BGC-823, HGC-27, MGC-803, and SGC-7901	Neutrophils	Induce autophagy and pro-tumor activation	[8]
	GKN1	HFE-145	AGS and MKN1	Inhibit gastric tumorigenesis	[47]
	CagA	CagA-expressing WT-A10	WT-10 and AGS	Involved in the development of extragastric disorders associated with CagA-positive *H. pylori* infection	[48]
	Apolipoprotein E	TAM	MFC and MGC-803	Promote cell migration	[49]
	MET	*H. pylori*-infected AGS	TAM	Promoted tumor growth and progression	[50]
	UBR2	p53^−/−^mBMMSC	p53^+/+^ mBMMSC and MFC	Promote cell proliferation, migration, and stemness	[51]
	TRIM3	Overexpressed MGC-803 and SGC-7901	MGC-803 and SGC-7901	Suppress gastric cancer growth and metastasis	[52]
miRNA	let-7 miRNA	AZ-P7a	/	Maintain cell malignance	[56]
	miR-423-5p	Overexpressed SGC-7901 and HGC-27	SGC-7901 and HGC-27	Promote cancer growth and metastasis	[54]
	miR-155-5p	Paclitaxel-resistant MGC-803R	Paclitaxel-sensitive MGC-803S	Promote EMT transition and chemoresistance	[55]
	miR-130a	SGC-7901	HUVECs	Promote angiogenesis and tumor growth	[57]
	miR-27a	SGC-7901	CCC-HSF-1	Promote transformation into cancer-associated fibroblasts	[23]
	miR-451	MKN45	Th17	Increase Th17 differentiation	[58]
	miR-21-5p	MGC-803, MKN-45, HGC-27, and SGC-7901	PMC and HMrSV5	Induce MMT and promote tumor peritoneal metastasis	[59]
	miR-21	TAM	BGC-823	Contributes to cell proliferation	[60]
	miR-21	TAM	MFC and MGC-803	Confer cisplatin resistance	[61]
	miR-221	GC-MSC	HGC-27	Promote cell proliferation and migration	[62]
	miR-221	BM-MSCs	BGC-823 and SGC-7901	Enhance cell proliferation, migration, invasion, and adhesion to the matrix	[63]
lncRNA	ZFAS1	BGC-823	MKN-28	Enhance cell proliferation and migration	[64]
circRNA	ciRS-133	SGC-7901	3T3L1	Promote differentiation into brown-like cells	[68]

### Exosomal proteins in GC

Proteins are one of the major components of exosomes. Most of the proteins in exosomes are ubiquitous regardless of cell types such as Ras-related protein and cytoskeletal proteins like tubulin, whereas some proteins in exosomes are cell type-specific. Initial proteomic studies revealed that tumor cells derived exosomes contain proteins including receptor proteins, heat shock proteins, fibronectin, integrin and others. In particular, several exosomal proteins have been reported to be implicated in GC progression. TGF-β1, an immunosuppressive cytokine produced by both tumor cells and immune cells, was found in plasma exosomes from GC patients and exosomal TGF-β1 level was correlated with lymphatic metastasis [43]. Exosomal TGF-β1 was capable of inducing the differentiation of regulatory T cells (Treg), which helps GC cells to evade the immune surveillance of the host. In addition, the work from Gu et al. showed that TGF-β transferred by GC cells derived exosomes interacted with TGF-βR1 in human umbilical cord-derived MSCs (hucMSCs), resulting in the activation of Smad pathway and the subsequent differentiation of hucMSCs to cancer-associated fibroblasts [44]. Bone morphogenetic protein 2 (BMP2), also belonging to the TGF-β superfamily, was reported to be highly expressed in GC cells derived exosomes [45]. They further demonstrated that exosomal BMP2 activated PI3K/AKT and MEK/ERK signaling pathways and induced pericyte-fibroblast transition, which was reversed by BMP pathway inhibitor. EGFR was also contained in GC cells derived exosomes and could be delivered into the liver and integrated on the plasma membrane of liver stromal cells. Exosomal EGFR suppressed miR-26a/b expression and then increased the expression of hepatocyte growth factor (HGF). The paracrine HGF bound to c-MET receptor on the migrated cancer cells to facilitate their landing and proliferation, which creates a favorable microenvironment for liver metastasis of gastric cancer cells [46].

Human gastrokine 1 (GKN1) is a protein that plays an important role in maintaining mucosal homeostasis. Yoon and colleagues demonstrated that GKN1 was secreted in exosomes and could be internalized by the gastric epithelium, thereby preventing the proliferation of malignantly transformed cells [47]. This specific exosomal transfer of GKN1 may be an important mechanism for the self-protection of human body against gastric tumorigenesis. Another protein CagA, encoded by cytotoxin-associated gene A, is a major virulence factor of *Helicobacter pylori (H. pylori)*. Shimoda et al. found that CagA protein was present in serum-derived exosomes from 4 GC patients infected with CagA-positive *H. pylori* [48]. The morphological changes of gastric epithelial cells and gastric cancer cells induced by CagA-containing exosomes indicated that exosomes-delivered functional CagA into cells may be involved in the development of extragastric disorders associated with CagA-positive *H. pylori* infection.

In a study by Zheng and collaborators [49], apolipoprotein E (ApoE) was identified as a highly specific protein in M2 macrophages-derived exosomes by mass spectrometry. The exosomes-mediated transfer of functional ApoE protein from tumor-associated macrophages to tumor cells promoted the migration of GC cells by triggering the activation of PI3K/Akt signaling pathway. In turn, GC cells could also deliver exosomal proteins to macrophages. Mesenchymal-epithelial transition factor (MET), highly expressed in exosomes from *H. pylori*-infected GC cells, could be delivered to and internalized by macrophages to educate them towards a pro-tumorigenic phenotype [50]. Recently, Zhang et al. performed a proteomic analysis of exosomes from three gastric cancer cell lines and identified high mobility group box-1 (HMGB1) protein as a key factor that participated in the promotion of pro-tumor activation of neutrophils [5]. They found that HMGB1 was transported by GC-derived exosomes to activate NF-κB pathway through interaction with TLR4, resulting in an increased autophagic response in neutrophils and in turn, promoting gastric cancer cell migration.

Ubiquitin protein ligase E3 component n-recognin 2 (UBR2) was found to be enriched in exosomes from p53 deficient mouse bone marrow MSC (p53^−/−^ mBMMSC) by Mao and colleagues [51]. They confirmed that UBR2 could be internalized into p53 wild-type mBMMSC (p53^+/+^ mBMMSC) and murine foregastric carcinoma (MFC) cells and increased the expression of UBR2 in these cells, which promoted gastric cancer growth and metastasis by activating wnt/β-catenin pathway. The expression of tripartite motif-containing 3 (TRIM3), a member of TRIM subfamily of the RING-type E3 ubiquitin ligases, was found to be lower in the serum exosomes of GC patients than that in the serum exosomes of healthy controls as reported by Fu et al. [52]. They also demonstrated that exosomes-mediated transfer of TRIM3 protein could suppress gastric cancer growth and metastasis through the regulation of stem cell factors and epithelial-to-mesenchymal (EMT) regulators.

### Exosomal microRNAs in GC

Numerous studies have analyzed the presence of genetic materials (including DNA and RNA) in exosomes. Among them, microRNAs are best described by far. The presence of small non-coding RNAs in exosomes from gastric cancer cells was identified by deep sequencing as reported by Ren and coworkers. MiR-100 and miR-148a were identified with aberrantly higher expression in GC cells derived exosomes compared to normal cells derived exosomes, which was further validated by quantitative reverse transcription polymerase chain reaction [53]. Yang et al. demonstrated that miR-423-5p enriched exosomes could be internalized into GC cells, which enhanced cell proliferation and migration by inhibiting the expression of suppressor of fused protein (SUFU) [54]. Recently, another miRNA contained in GC cells derived exosomes was revealed by Wang and colleagues. They reported that miR-155-5p could be shuttled by exosomes from paclitaxel-resistant GC cells to paclitaxel-sensitive GC cells, resulting in strengthened capacity of reducing paclitaxel chemosensitivity in the parental cells [55]. Intriguingly, the release of exosomes enriched with tumor suppressive miRNAs may help maintain the malignant phenotype of GC cells. For instance, Oshima et al. demonstrated that the exosomal release of let-7 miRNAs into the extracellular environment resulted in the maintenance of tumorigenic phenotype and metastatic potential of GC cells [56].

Exosomes secreted by GC cells can also transfer selectively packaged miRNAs to other cells in tumor microenvironment. The uptake of GC cell-secreted exosomal miR-130a inhibited *c-myb* gene expression in vascular endothelial cells, promoting angiogenesis and tumor growth [57]. Wang and collaborators observed that exosomes released by GC cells could induce a transition of fibroblasts into CAFs through miR-27a, which in turn promoted the proliferation, motility and metastasis of GC cells [23]. Liu et al. reported that the redistribution of GC cells derived miR-451 to infiltrated T cells could enhance T helper 17 cell differentiation by activating mTOR [58]. In addition, another miRNA originated from GC cells derived exosomes, miR-21-5p, could induce mesothelial-to-mesenchymal transition of peritoneal mesothelial cells and promote gastric cancer peritoneal dissemination by targeting SMAD7 [59].

Emerging studies suggest that exosomes derived from non-tumor cells could deliver miRNAs to GC cells during tumor progression. Wang and colleagues demonstrated that exosomal miR-21 could be directly transferred from macrophages to GC cells to promote cell migration and suppress cell apoptosis [60]. This observation is also supported by another study, in which Zheng et al. reported that M2 macrophages-derived miR-21 conferred cisplatin resistance in gastric cancer cells [61]. Exosomal miR-21 inhibited programmed cell death 4 (PDCD4) gene expression [60] and activated PI3K/AKT signaling pathway by down-regulation of PTEN [61]. In addition, the study by Wang et al. revealed that miR-221 enriched in exosomes from gastric cancer tissue-derived mesenchymal stem cells (GC-MSCs) promoted GC cell proliferation and migration [62]. This finding was recently corroborated in an independent study, in which Ma and coworkers showed that bone marrow mesenchymal stem cells (BM-MSCs) derived exosomes could deliver miR-221 to promote GC cell proliferation and migration [63].

### Exosomal lncRNAs and circRNAs in GC

In addition to miRNAs, exosomes are now known to contain several other species of non-coding RNAs, such as lncRNAs and circRNAs, which also exert important regulatory functions in GC. A pioneering study revealed that long intergenic non-protein-coding RNA 152 (LINC00152) was present in exosomes and was highly expressed in the plasma of GC patients [7]. The presence of LINC00152 in exosomes may protect them from RNase degradation. The study from Pan et al. demonstrated that lncRNA ZFAS1 was enriched in exosomes from the serum of GC patients and exosomes-mediated transfer of ZFAS1 could enhance GC cell proliferation and migration [64]. In addition, Zhao et al. validated the existence of another lncRNA HOTTIP in exosomes from the plasma of GC patients [65], which were remarkably upregulated in GC compared to healthy individuals. Similarly, exosomal lncUEGC was confirmed to be remarkably up-regulated in early gastric cancer patients compared to healthy controls, indicating its capability as a sensitive and stable biomarker for early gastric cancer diagnosis [66]. Circ-KIAA1244 was recently identified in a circRNA microarray and found to be downregulated in the plasma of GC patients. The comparable expression level of circ-KIAA1244 was also detected in exosomes from the plasma of GC patients, suggesting that circ-KIAA1244 might be transmitted to plasma via exosomes [67]. CircRNAs delivered by GC cells derived exosomes also exert regulatory roles in other cells. Zhang and collaborators suggested that exosomal circRNA ciRS-133 secreted from GC cells promoted the differentiation of pre-adipocytes to brown-like cells by functioning as a miRNA-133 sponge and activating PRDM16 expression [68].

## Potential application of exosomes in GC

### Exosomes as biomarkers in GC

The unique expression pattern and the relative stability of its contents have made exosomes a new candidate for tumor liquid biopsy. Increasing studies have shown that exosomes may have a great potential to serve as biomarkers for the early diagnosis, the prediction of prognosis, and the evaluation of therapy effect in GC (Table 2).

**Table 2:** Exosomes extracted from biofluids as diagnostic and prognostic biomarkers for GC

Cargo type	Exosomal cargo	Biofluids	Extraction method	Identification method	Method	Clinical value in GC	Reference
Protein	BARHL2	Gastric juice	Commercial kit	Not shown	qRT-PCR	BARHL2 methylation yielded an AUC of 0.923 with 90% sensitivity and 100% specificity	[69]
	GKN1	Serum	Commercial kit	TEM	ELISA	Discriminate GC patients from healthy individuals (AUC = 1.00), patients with hepatocellular (AUC = 1.00) and colorectal carcinomas (AUC = 0.98)	[47]
	TGF-β1	Plasma	Differential centrifugation	Western blot, immuno-EM and NTA	ELISA	Associated with lymph node metastasis	[43]
	TRIM3	Serum	Commercial kit	TEM and NTA	ELISA, western blot	Downregulated in GC and may serve as a biomarker for GC diagnosis	[52]
miRNA	miR-423-5p	Serum	Commercial kit	TEM, NTA and western blot	qRT-PCR	Higher diagnostic efficiency than CEA and CA-199; indicator for poor prognosis	[54]
	miR-451	Serum	Commercial kit	Not shown	qRT-PCR	indicate poor prognosis of GC patients	[58]
	miR-217	Plasma	Differential centrifugation	Not shown	qRT-PCR	Higher expression in GC patients	[71]
	miR-23b	Plasma	Ultracentrifugation	TEM	miRNA microarray, qRT-PCR	Predicts the recurrence and prognosis of GC patients in each tumor stage	[74]
	miR-19b-3p and miR-106a-5p	Serum	Commercial kit	TEM and western blot	qRT-PCR	Related to GC lymphatic metastasis and TNM stage; more accurate than AFP and CA19–9 for GC diagnosis	[70]
	miR10b-5p, miR195-5p, miR20a-3p, and miR296-5p	Serum	Commercial kit	Not shown	miRNA microarray, qRT-PCR	Elevated expression in GC patients	[24]
	miR-424-5p and miR-590-3p	Serum	Commercial kit	TEM, NTA and western blot	miRNA profiling,qRT-PCR	Effective biomarker for diagnosing the stage of GC progression	[72]
	miR-21 and miR-1225-5p	Peritoneal lavage fluid	Differential centrifugation	Not shown	miRNA microarray, qRT-PCR	Biomarker for the prediction of peritoneal dissemination	[73]
lncRNA	lncUEGC1	Plasma	Serial centrifugation and discontinuous iodixanol gradient	TEM, NTA and western blot	RNA sequencing, qPCR	Potential early GC biomarker with higher diagnostic accuracy than CEA	[66]
	HOTTIP	Serum	Ultracentrifugation	Not shown	qRT-PCR	Higher diagnostic capability than CEA, CA 19–9 and CA72–4; correlated with poor overall survival	[65]
	ZFAS1	Serum	Commercial kit	TEM, NTA and western blot	qRT-PCR	Higher levels in GC patients; associated with lymph node metastasis and TNM stage	[64]
	LINC00152	Plasma	Commercial kit	TEM	qRT-PCR	Higher levels in GC patients	[10]
circRNA	circ-KIAA1244	Plasma	Commercial kit	Not shown	circRNA microarray, qRT-PCR	Potential diagnostic and prognostic biomarker for GC	[67]

#### Exosomal DNA

Yamamoto et al. isolated exosomes from the gastric juice of 20 GC patients and 10 non-GC controls to detect the status of BARHL2 gene methylation [69]. The methylated level of BARHL2 gene yielded an area under the receiver operating characteristic curve (ROC) of 0.923 with 90% sensitivity and 100% specificity to discriminate GC patients from non-GC controls. Therefore, this gastric juice-derived exosomal DNA could be an excellent candidate for GC diagnosis in the future. However, further studies with a large cohort of samples are warranted to verify the above observation.

#### Exosomal protein

Several exosomal proteins have been demonstrated to possess diagnostic value in GC as detected by ELISA. Yoon and collaborators successfully detected GKN1 in serum derived exosomes that had been heated at 70 °C for 10 min but not in the unheated samples. Serum GKN1 levels in GC patients were significantly lower than those in healthy individuals. The serum level of GKN1 could discriminate GC patients from healthy controls and subjects with atrophic gastritis. Moreover, the serum GKN1 level was able to discriminate GC patients from patients with hepatocellular and colorectal carcinomas, suggesting its potential as GC-specific diagnostic marker [47]. In addition, Yen et al. revealed that increased exosomal TGF-β1 expression level was correlated with advanced stages and lymph node metastasis, according to a detection enrolling 61 GC patients [43]. Moreover, Fu and colleagues revealed that the levels of TRIM3 protein in the serum exosomes of GC patients were significantly lower than that in healthy controls [52]. These observations indicate the potential of the above proteins as future diagnostic and prognostic biomarkers for GC.

#### Exosomal miRNA

The recent studies also indicate that exosomal miRNAs may serve as potent biomarkers for GC. Microarray profiles revealed that miR-19b-3p and miR-106a-5p were significantly upregulated in the serum exosomes of GC patients. Interestingly, a miRNA signature combined by the above two miRNAs provided a remarkable area under curve (AUC) value of 0.814 in discriminating GC patients from healthy volunteers. Furthermore, this miRNA panel exhibited a higher diagnostic sensitivity and specificity than AFP and CA-199 [70]. Another study identified 6 miRNAs that were significantly upregulated in the serum of GC patients, whereas only four of them (miR-10b-5p, miR-195-5p, miR-20a-3p, and miR-296-5p) showed significantly elevated expression in serum exosomes [24]. In addition, the deregulation of miR-217 was found in plasma exosomes and could be used as a biomarker for GC diagnosis and classification [71]. Cancer stem-like cells (CSCs) derived miRNA signature may also provide biomarkers for GC diagnosis as these cells are recognized as the initiating cells for carcinogenesis. Sun et al. identified 11 differentially expressed miRNAs in exosomes from gastric CSCs by microarray analysis, including 6 up-regulated miRNAs (miR-1290, miR-1246, miR-628-5p, miR-675-3p, miR-424-5p, and miR-590-3p) and 5 down-regulated miRNAs (let-7b-5p, miR-224-5p, miR-122-5p, miR-615-3p, and miR-5787). Among them, serum exosomal miR-424-5p and miR-590-3p exhibited most differential expression in the validation study [72]. Peritoneal metastasis is associated with poor prognosis of GC patients. Tokuhisa and colleagues assessed the diagnostic potential of exosomal miRNA profiles in peritoneal fluid for the prediction of peritoneal dissemination. Five exosomal miRNAs were detected with high expression in malignant ascites, peritoneal lavage fluids, and GC cell culture media. Among them, miR-21 and miR-1225-5p were confirmed to be associated with serosal invasion in GC, providing biomarkers for early diagnosis of peritoneal dissemination in GC [73]. Recently, Yang et al. found that the levels of exosomal miR-423-5p were elevated in the serum of GC patients and significantly correlated with lymph node metastasis. Subsequent ROC curve analysis showed a higher diagnostic value of serum exosomal miR-423-5p than CEA and CA-199. Furthermore, high level of serum exosomal miR-423-5p predicted poor prognosis in GC patients [54]. Another exosomal microRNA, miR-451, also displayed the ability of predicting prognosis in post-surgery GC patients, with high miR-451 group showing a significantly poorer prognosis of 5-year survival compared to low miR-451 group [58]. Moreover, Kumata et al. identified exosomal miR-23b as an independent prognostic factor for overall survival and disease-free survival at each tumor stage, providing predictive biomarker for the recurrence and prognosis of GC in patients at all stages [74]. Collectively, these results suggest that exosomal miRNAs could not only serve as novel non-invasive diagnostic, but also promising prognostic biomarkers for GC.

#### Exosomal lncRNA and circRNA

Exosomal lncRNAs and circRNAs have also been unveiled as diagnostic markers for GC. The study by Li et al. revealed that the plasma LINC00152 levels were significantly elevated in GC patients compared with healthy controls. In addition, LINC00152 levels in preoperative plasma samples were lower than those in postoperative ones. Importantly, no differences of LINC00152 levels were shown between plasma and plasma exosomes, indicating that LINC00152 mainly exists in exosomes [7]. Pan and colleagues found that exosomal ZFAS1 levels were elevated in GC patients and associated with TNM stage and lymphatic metastasis [64]. In a recent study, Lin et al. revealed that lncUEGC1 was significantly upregulated in plasma exosomes from stage I GC patients compared to healthy controls. Plasma exosomal lncUEGC1 showed better diagnostic value in distinguishing stage I GC patients from healthy individuals (AUC = 0.85) or premalignant lesions (AUC = 0.84) than serum CEA, indicating that exosomal lncUEGC1 may be a promising candidate for highly sensitive non-invasive biomarkers for GC diagnosis [66]. In addition, the expression of serum exosomal HOTTIP was upregulated in GC and significantly correlated with invasion depth and TNM stage. Exosomal HOTTIP demonstrated a higher diagnostic accuracy than conventional biomarkers CEA, CA19–9 and CA72–4. Moreover, increased exosomal HOTTIP levels significantly correlated with poor overall survival, making exosomal HOTTIP an independent prognostic factor in GC patients [65]. More recently, circRNA expression profile analysis showed lower expression of circ-KIAA1244 in GC tissues and plasma samples. The decreased expression of plasma circ-KIAA1244 was negatively correlated with TNM stage and lymphatic metastasis, and a shorter overall survival. Furthermore, circ-KIAA1244 could also be detected in plasma exosomes, with its expression level comparable to that in plasma, suggesting that circ-KIAA1244 is encapsulated in exosomes in the plasma of GC patients [67].

### Exosomes as therapeutic targets in GC

#### Exosome-based immunotherapy

The application of exosomes in GC therapy is still at an early stage. Exosomes were first used for immunotherapy of GC in 2011. Zhong et al. demonstrated that after heat treatment, exosomes from malignant ascites of GC patients were able to promote dendritic cell maturation and induce a tumor-specific cytotoxic T lymphocyte response [75]. These observations suggest that the exposure to heat stress could improve the immunogenicity of exosomes isolated from malignant ascites of GC patients, which represents an effective tumor vaccine.

#### Exosomes as new drug targets

The important roles of exosomes in GC suggest that it may be exploited as a therapeutic target. Proton-pump inhibitors (PPIs) have been shown to reduce gastric acid production and facilitate the effects of anti-cancer drug in GC cells. Guan et al. recently demonstrated that PPIs could inhibit the release of exosomes from GC cells and antagonizing the induction of CAFs by GC cells, suggesting that PPIs may be of potential value as a therapeutic tool for gastric cancer treatment through the inhibition of exosome release [76].

#### Exosomes as novel modes of drug delivery

Exosomes have been used to deliver biological molecules and chemotherapeutic drugs for cancer therapy. For example, exosomes are considered as efficient delivery vehicles for RNA-based therapeutic strategies. Anti-miR-214 loaded in exosomes reversed the resistance of GC cells to cisplatin, which might serve as an alternative for the treatment of cisplatin-refractory GC [77]. Zhang et al. showed that HGF siRNA loaded in exosomes can be transported into GC cells to suppress their proliferation and migration. Importantly, these exosomes were able to deliver HGF siRNA in vivo, inhibiting tumor growth and angiogenesis in nude mice [78]. Wang and colleagues suggested that macrophages derived exosomes could be used as vehicles to transfer miR-21 inhibitor into GC cells, reducing cell migration and inducing cell apoptosis [60]. Anti-cancerous proteins could also be delivered via exosomes and exert tumor-inhibitory effects. Yoon et al. demonstrated that exosomes carrying tumor suppressive GKN1 protein were secreted and internalized in the gastric epithelium to inhibit gastric tumorigenesis, indicating its clinical application in GC treatment [47]. Similarly, exosomes-based delivery of TRIM3 protein has been shown to suppress GC growth and metastasis in vitro and in vivo, thus providing a novel avenue for GC therapy [52].

## Conclusions

The current researches on exosomes have reshaped our understanding of exosome in cancer biology and provide new targets for cancer diagnosis and therapy. Exosomes have gained extensive attentions in cancer due to its multifaceted roles such as reprogramming tumor behaviors, remodeling tumor microenvironment and conferring therapy resistance. The existing studies have partially unraveled the mechanisms of actions of GC-relevant exosomes, which mainly could be attributed to the specific cargos that they bear. In particular, there seems to be a bidirectional transfer of molecules between GC cells and the stromal cells in tumor microenvironment, helping establish pre-metastatic niche and develop therapy resistance. Moreover, exosomes loaded with specific bioactive molecules in the circulation show desirable diagnostic value, reflecting the stage of GC progression and metastasis, as well as potential capability of predicting prognosis for GC patients. Exosomes-based therapeutics in GC have also shown great promise. Blocking GC cells derived exosome release, utilizing malignant ascites derived exosomes as vaccines, delivering tumor suppressive molecules and drugs alone or in conjunction with traditional therapies provide new strategies for GC treatment. Despite increased focus on exosomes in GC, there are still several challenging problems to be addressed before the use of exosomes in the clinical management of GC, including the depiction of detailed mechanisms for the aforementioned roles of exosomes in GC, the establishment of standardized methods for the isolation, quantification, and analysis of exosomes in the body fluids of GC patients, the detection of GC-specific exosomal DNA, RNA species, or proteins in the circulation, and the identification of exosomes from the optimal donor cells for drug loading and large-scale preparation, storage, and formulation. Therefore, more efforts still need to be devoted to better understand the roles and mechanisms of action of exosomes in GC and to develop exosomes-based clinical regimens for GC diagnosis, prognosis and therapy.

## Abbreviations

5-FU: 5-fluorouracil
ApoE: apolipoprotein E
AUC: area under curve
BM-MSCs: bone marrow mesenchymal stem cells
BMP-2: bone morphogenetic protein 2
CAFs: cancer-associated fibroblasts
Cag A: cytotoxin associated gene A
CircRNA: circular RNA
CSCs: cancer stem cells
EGFR: epidermal growth factor receptor
EMT: epithelial-mesenchymal transition
ESCRT: endosomal sorting complex required for transport
GC: gastric cancer
GC-MSCs: gastric cancer tissue-derived mesenchymal stem cells
GKN1: gastrokine 1
*H. pylori*: Helicobacter pylori
HGF: hepatocyte growth factor
HMGB1: high mobility group box-1
LINC00152: long intergenic non-protein-coding RNA 152
MET: mesenchymal-epithelial transition factor
MFC: murine foregastric carcinoma
MMT: mesothelial-to-mesenchymal transition
MSCs: mesenchymal stem cells
MVBs: multivesicular bodies
PD-1: programmed death-1
PDCD4: programmed cell death 4
PD-L1: programmed cell death ligand 1
PMC: peritoneal mesothelial cell
PPIs: proton-pump inhibitors
ROC: receiver operating characteristic curve
SUFU: suppressor of fused protein
TAMs: tumor-associated macrophages
TANs: tumor-associated neutrophils
TGF-β1: transforming growth factor-β 1
TGF-βR1: transforming growth factor-β receptor 1
Treg: regulatory T cells
TRIM3: tripartite motif-containing 3
UBR2: ubiquitin protein ligase E3 component n-recognin 2
